# Parents’ Attitudes toward Clinical Genetic Testing for Autism Spectrum Disorder—Data from a Norwegian Sample

**DOI:** 10.3390/ijms18051078

**Published:** 2017-05-18

**Authors:** Jarle Johannessen, Terje Nærland, Sigrun Hope, Tonje Torske, Anne Lise Høyland, Jana Strohmaier, Arvid Heiberg, Marcella Rietschel, Srdjan Djurovic, Ole A. Andreassen

**Affiliations:** 1 NORMENT, KG Jebsen Centre for Psychosis Research, University of Oslo, Oslo 0424, Norway; ternae@ous-hf.no (T.N.); sighop@ous-hf.no (S.H.); srdjan.djurovic@medisin.uio.no (S.D.); ole.andreassen@medisin.uio.no (O.A.A.); 2 Division of Mental Health and Addiction, Oslo University Hospital, Oslo 0315, Norway; 3 Department of Medical Genetics, Oslo University Hospital, Oslo 0424, Norway; arvhei@ous-hf.no; 4 NevSom, Department of Rare Disorders and Disabilities, Oslo University Hospital, Oslo 0424, Norway; 5 Autism Society Norway, Oslo 0609, Norway; 6 Department of Neurohabilitation, Oslo University Hospital, Oslo 0424, Norway; 7 Regional Centre for Child and Youth Mental Health and Child Welfare, Department of Mental Health, Faculty of Medicine and Health Sciences, Norwegian University of Science and Technology, Trondheim 7491, Norway; anne.lise.hoyland@ntnu.no; 8 Department of Pediatrics, St. Olavs Hospital, Trondheim University Hospital, Trondheim 7006, Norway; 9 Department of Genetic Epidemiology in Psychiatry, Central Institute of Mental Health, Medical Faculty Mannheim, University of Heidelberg, Mannheim 68159, Germany; Jana.Strohmaier@zi-mannheim.de (J.S.); Marcella.Rietschel@zi-mannheim.de (M.R.); 10 Division of Mental Health and Addiction, Vestre Viken Hospital Trust, Drammen 3004, Norway; tonje.torske@vestreviken.no; 11 Department of Clinical Science, University of Bergen, Bergen 5021, Norway

**Keywords:** ASD, autism, Asperger syndrome, clinical genetic testing, parents, attitudes, ethics, genetic counselling

## Abstract

Clinical genetic testing (CGT) of children with autism spectrum disorder (ASD) may have positive and negative effects. Knowledge about parents’ attitudes is needed to ensure good involvement of caregivers, which is crucial for accurate diagnosis and effective clinical management. This study aimed to assess parents’ attitudes toward CGT for ASD. Parent members of the Norwegian Autism Society were given a previously untested questionnaire and 1455 answered. Linear regression analyses were conducted to evaluate contribution of parent and child characteristics to attitude statements. Provided it could contribute to a casual explanation of their child’s ASD, 76% would undergo CGT. If it would improve the possibilities for early interventions, 74% were positive to CGT. Between 49–67% agreed that CGT could have a negative impact on health insurance, increase their concern for the child’s future and cause family conflicts. Parents against CGT (9%) were less optimistic regarding positive effects, but not more concerned with negative impacts. The severity of the children’s ASD diagnosis had a weak positive association with parent’s positive attitudes to CGT (*p*-values range from <0.001 to 0.975). Parents prefer that CGT is offered to those having a child with ASD (65%), when the child’s development deviates from normal (48%), or before pregnancy (36%). A majority of the parents of children with ASD are positive to CGT due to possibilities for an etiological explanation.

## 1. Introduction

Autism spectrum disorder (ASD) is a set of heterogeneous conditions, characterized by early-onset difficulties in social communication and unusually restricted, repetitive behavior and interests [1]. The worldwide prevalence is about 1%, with a high global disease burden [2]. In Norway, the cumulative incidence for 11-year-olds has recently been estimated to be 0.8% [3].

ASD is among the most heritable neurodevelopmental conditions [4]. The recurrence risk for a sibling of an affected child is about 10–20%, which increases to 30–50% if the child has more than one sibling with ASD [5]. The etiology is still largely unknown, although it is clear that environmental factors are involved in addition to genetic susceptibility [1]. The etiology of ASD is very heterogeneous [6], and the search for genes that can explain the pathology is ongoing.

Recent advances in genetic technology have increased the diagnostic yield to 30–40% in clinical testing [7], and genetic testing is increasingly being used in the clinics, although not as a diagnostic tool [8]. In Norway, clinical genetic testing (CGT) is performed upon request by specialists in medical genetics, neurology or pediatrics and requires good phenotypic description. In cases of developmental delay and intellectual disability, as well as at least some features of ASD, chromosome 16p11.2 deletion syndrome is specifically tested for. Other copy number variants (CNVs) of diagnostic yield are emerging. A recent study on the use of ultra-high resolution chromosomal microarray analysis (CMA) found 15q11.2 BP1–BP2 microdeletion as the most common cytogenetic finding in those with ASD presenting for genetic services [9].

Meaningful interventions exist for ASD, but approved medications that target the core symptoms are absent. Finding effective treatments is regarded as the most important goal of ASD research [10], and the identification of a genetic etiology is a first step in the development of an individualized medical approach for these patients [5].

Towards the achievements of a new era of precision medicine [11], essential non-technological issues must be clarified. These issues include respecting the patients’ autonomy and privacy and their need for protection from discriminations [12]. Good involvement of caregivers is needed to improve the accuracy of diagnosis, and to enable effective clinical management, tailored education [13,14], and genetic counseling, which involves complicated issues [15,16,17] that require a high degree of ethical reflection [15] informed by up-to-date knowledge about technological advancements [18,19] as well as of the views of those involved.

According to a recent report, professionals believe that genetic testing could improve the possibility for early intervention, enable the prevention of specific comorbid diseases, or relieve guilt [20]. Clarifying the role of specific genetic factors may contribute to parents’ understanding of why their child has ASD [5]. Clinical relevance of genetic factors may include impact on the diagnostic process [15,21,22], on pharmacological and behavioral treatment [23], on adequate planning of actions [15] and on preparing parents for a life with an ASD child [22].

However, knowledge of specific genetic factors may also be potentially harmful, depending on the characteristics and roles of the genes involved, the family situation and clinical relevance of the genetic factors. A test showing that a child inherited a disease-causing gene may induce feelings of guilt and worry while a de novo mutation may relieve the parents from feelings of responsibility [21,24]. Genetic testing may also cause family conflicts [25]. Feelings of guilt and family conflicts are common in monogenic disorders [26,27]. The likelihood of recurrence of ASD in the family involved, i.e., the risk of getting another child with ASD [28,29,30], is also relevant for whether knowledge of specific genetic factors may be beneficial or harmful.

Current knowledge of parents´ attitudes toward CGT for ASD is limited. One study [31] found that more than 90% of the parents agreed that genetic testing is useful in health care, and that 86% of the parents are interested in finding out if genetic factors are causal in their child’s ASD. Another study [32] shows that 80% of parents were interested in a genetic risk assessment test for ASD in an undiagnosed younger sibling. Both studies found that parents held a belief that the genetic test result could allow for closer monitoring and earlier access to evaluation and intervention. However, these studies included a small number of parents. Early identification is important for access to specialized evidence-based interventions [33], and prolonged diagnostic process is associated with stress in parents [34]. Parental stress or psychological well-being is not significantly different in parents of children across the ASD sub-diagnoses of infantile autism and Asperger syndrome [35]. However, we previously found very small significant differences in attitudes toward genetic research between parents of children with autism and parents of children with Asperger syndrome [36]. 

Perceived recurrence risk is found to influence reproductive decisions [37]. A survey of parents of children with fragile X syndrome (FXS) found that 59% indicated that the diagnosis affected their decision to have another child greatly while the rest was less affected. Those who indicated they were unaffected had already decided not to have more children [38,39]. A review found that concerns about increased risk of having a child with a given genetic condition influences decisions to undergo prenatal testing [40]. To the extent that these findings are applicable to ASD, they suggest that the reproductive decisions of parents of ASD individuals may be influenced by their perceived risk [39].

Genetic test results are also believed to reinforce stigmatization and discrimination, both by society in general and by insurance companies [25]. The stigma attached to mental illness is claimed to always lead to stress through discrimination in academic settings, in work, and in public, although often unintended [41]. Advocates for ASD fear the possible outcomes of genetic prenatal testing or treatment and argue that aggressively seeking interventions devalues autistic traits and tendencies [42]. Adults with ASD fear that people with ASD traits eventually will be eliminated through prenatal testing and selective abortion [42]. Despite genetic non-discrimination acts in most western countries [21,25], people with a disease mutation may be offered less favorable health insurance [25,43]. In some countries, it is explicitly allowed to use genetic risk information when the insurance payment exceeds a certain level [44,45].

To the best of our knowledge, most findings with respect to attitudes in the field are generated from small samples or samples that may be skewed due to selection bias or self-reference. Thus, there is a need for larger empirical investigations on parents’ attitudes and outlooks of CGT in ASD. The objective of the current study was to describe and explore parents’ attitudes to CGT for ASD. Our first hypothesis was that (1) parents would be positive toward CGT for ASD, due to learning more about their child’s genetic risk and etiology of confirmed ASD. We also hypothesized that (2) small differences in attitudes to CGT exist between parents of children with autism and parents of children with Asperger syndrome. Health or life insurance discrimination is a potential negative effect of CGT for ASD risk. Thus, the third hypothesis was that (3) discrimination from insurance companies would be a reason to be opposed to CGT. Early diagnosis is important for early access to specialized evidence-based interventions and our fourth hypothesis was that (4) parents of children with ASD would prefer CGT for ASD risk to be offered as early as possible, i.e., during pregnancy. As a corollary, we also hypothesized that (5) parents of children with ASD would prefer CGT for ASD risk to be offered to all those interested, i.e., those doubting they are able to care for a child with ASD.

## 2. Results

### 2.1. Participants: Sociodemographic

As seen in Table 1, mean age of the parents was 46.7 years, and the mean age of their children with ASD was 16.5 years. Of the children with ASD, 81% were male and 19% were female, 49% had Asperger syndrome and 51% had infantile autism or atypical autism (infantile autism 38%, atypical autism 13%).

### 2.2. Possible Positive Effects of Clinical Genetic Testing

Proportions of participants agreeing to statements about expected positive and negative effects of CGT for ASD are presented in Table 2. Regression model summaries are listed in Table S1a,b.

Three quarters of the participants would only do CGT if it could contribute to explaining the etiology of ASD in their child (76%) and if the CGT could improve the possibilities of planning for good interventions and treatments (75%). Half of the respondents would do CGT only if it has a direct relevance for treatment or intervention (53%). A total of 40% would do CGT if it enabled them to prevent having further children with ASD, and 24% agreed that CGT is important for family planning.

Some parent and child characteristics had a small but significant influence on opinions regarding positive effects of clinical genetic testing. Parents of children with autism differed from parents of children with Asperger syndrome on how CGT is important for etiological explanation, for recurrence prevention and for family planning. Parents of children with autism parents agreed significantly more to the statements than parents of children with Asperger syndrome in all three models (*p* < 0.001). In a family planning model, age of parent contributed significantly (*p* = 0.007). Agreement to the family planning statement increases by age of the parent. The variances explained in the significant models are, however, small (*p*-values range from <0.001 to 0.009; *R*^2^ range from 0.018 to 0.081). Regression model summaries are presented in Table S1a.

### 2.3. Possible Negative Effects of Clinical Genetic Testing

Two thirds (67%) of the parents agreed that a genetic test result showing risk for ASD will cause insurance company discrimination, and over half (56%) had increased concern for the child’s future as a consequence of CGT. Half (49%) of the parents agreed that genetic testing may cause family conflicts. We found no significant association between age, gender or ASD sub-diagnosis and opinions of insurance discrimination, parental concern or family conflicts (*p*-values range from 0.055 to 0.404) (Table S1b).

### 2.4. Management of Clinical Genetic Testing: Who and When?

The proportions of participants’ opinions concerning who should be offered CGT for ASD and when to offer CGT are listed in Table 3. Regression model summaries are listed in Table S2a,b.

On opinions about who should be offered CGT for ASD risk, 65% of the respondents agreed to those who already have an ASD child, 16% agreed to those doubting they are able to care for a child with ASD and 15% agreed to those worried about the fetus. A total of 11% of the respondents agreed that CGT should be offered to no pregnant women, and 10% agreed to all pregnant women.

Regression analyses of the parent and child characteristics (see Table S2a) show small but significant contributions from ASD sub-diagnoses in the parents of children with an ASD model and in those worried about the fetus model. Parents of children with autism agree more than parents of children with Asperger syndrome that parents of children with ASD and those worried about the fetus should be offered CGT for ASD risk (*p* < 0.001).

On items about time of testing, 48% agreed to do CGT when development deviates, 36% before pregnancy, 9% during pregnancy and 8% immediately after birth. Regression analyses of the parent and child characteristics (see Table S2b) show small but significant contributions from ASD sub-diagnosis in the before pregnancy model (*p* < 0.001) and the during pregnancy model (*p* = 0.015). Parents of children with autism parents agreed more than parents of children with Asperger syndrome in both models. The variances explained in the significant models are, however, small (*p* = 0.002; *R*^2^ = 0.017 and 0.018).

### 2.5. Participants Opposing Clinical Genetic Testing

One option on the question “When is the best time to offer genetic testing for ASD risk?” was “I am against genetic testing for ASD”, to which 9% of the participants agreed. Of those opposing CGT, 31% agreed that they would do testing only if it could help explain the cause of ASD, 42% if it has directly treatment-relevant consequences, and 35% if that early diagnosis based on CGT will improve the possibilities of planning for good interventions and facilitations (Table 4).

Parents opposing CGT differ significantly from parents not explicitly opposing all statements (*p*-values range from <0.001 to <0.01). We found the largest differences in scores on the statements of positive effects and the smallest differences on negative effects. See Table 4 for details.

## 3. Discussion

The main result of this study is that parents of ASD individuals have a clearly positive view on CGT for ASD. Etiological explanation is the most frequent effect of CGT that our respondents agreed to in our survey. This seems to confirm our hypothesis #1. The second positive effect was improvement in the possibilities of planning for good interventions and facilitations. These results are in line with findings from a study of professionals who also believe that genetic testing implies benefits for the affected families, e.g., a better possibility for early intervention, prevention of specific comorbid diseases, or relief of guilt [20]. The current findings seem to provide mixed support for routine CGT for ASD, and could help in designing such programs.

Nine percent of our respondents seem to be clearly against CGT. However, this seems mostly due to doubts about the positive effects and less due to fears of negative effects. The large agreement to positive effects of CGT among the responders (Table 4) may indicate that they will become in favor of CGT when clinical utility and treatment implications improve. Taken together, these findings seem to indicate rather robust positive attitudes toward CGT for ASD among parents of children with ASD. Furthermore, the findings also suggest that there are very small differences between parents of children with autism and parents of children with Asperger syndrome, supporting our hypothesis #2.

Skepticism against beneficial effects of CGT rather than fear of negative consequences seems to be a general finding of the current analysis. The parents’ attitudes could be interpreted as being cautious and focused on preventing negative consequences of introducing technological innovations into clinical practice prematurely, e.g., by giving inaccurate etiological diagnoses [46]. Their opinions seems to be in line with the recent guidelines of EuroGentest and the European Society of Human Genetics (ESHG) for diagnostic next-generation sequencing, which states that insufficiently validated tests present a threat to patients, and their use in a clinical diagnostic setting is unacceptable [47].

Discrimination from insurance companies is the negative effect of CGT most frequently stated in our sample. The difference between those in favor and those against CGT is small (see Table 4), supporting our hypothesis #3. Fear of discrimination from insurance companies may come as a surprise in the Norwegian context of our study. In Norway, genetic testing is covered by the compulsory state health insurance, and discrimination on the basis of genetic constitution is prohibited by law [48], as it is in most other western countries [21,25]. The UN Universal Declaration of the Human Genome and Human Rights also prohibit all forms of discrimination based on genetic characteristics [49]. However, the use of voluntary private health insurance (VPHI) has recently increased also among Norwegians, but VPHI typically do not cover treatments for psychiatric conditions [50]. Knowledge of risk is integral to the concept of insurance premiums [44] and some European countries explicitly allow the use of genetic risk information when the insurance payment exceeds a certain level, which usually applies for life insurance, illness/health insurance or insurance to protect income in the case of being disabled and unable to work [44,45]. Having a child with ASD is associated with long term sick leave, not being in the labor force and low income [51], and it seems prudent to be aware of issues of possible discriminations from health and life insurance companies both in relation to parents and to people with ASD.

While only 8% agree that it is best to offer CGT during pregnancy (i.e., prenatal diagnosis), half (48%) of the parents in our sample prefer CGT to be offered when a child shows behavioral or developmental challenges and more than a third (36%) before pregnancy. Hypothesis #4, saying that parents would prefer CGT as early as possible, i.e., during pregnancy, is thus refuted. In the event of a prenatal test for ASD, this result may indicate that the Norwegian practice of direct access to prenatal testing for Trisomies (21, 18, 13) for women 38 years or older, or in later pregnancies when one has a child with a severe disability, may be less relevant for parents of individuals with ASD. This may have consequences for genetic counselling practices of parents of children with ASD. Effective counselling may depend on professional exposure to individuals with ASD. In a Down syndrome (DS) context, it is claimed that what genetic counselors believe to be the most salient information to discuss with parents differs based on whether they practice in the prenatal or postnatal setting [52], which may be a point to consider in ASD as well.

We found that more than half (65%) of the parents of children with ASD prefer CGT to be offered to those already having a child with ASD. This contradicts our hypothesis #5 that parents would prefer all those interested, i.e., those doubting that they are able to care for a child with ASD, to be offered CGT. Fifteen percent of the respondents support our hypothesis #5, and 36% prefer CGT before pregnancy. Although not specified, testing before pregnancy is plausibly interpreted as carrier testing in parents, which is not likely in a complex disorder such as ASD. This may reflect a lack of knowledge in parents, but may as well be due to the questionnaire that asked to imagine the existence of a test saying “something about the risk of ASD”. However, parents’ recurrence risk perceptions have shown to be inaccurately high and to influence reproductive decisions [39], and our finding may be interpreted in line with this.

We found that 40% in our sample would do a CGT if it gave them the possibility to prevent having more children with ASD. This may be interpreted as our sample preferring CGT when a child shows behavioral or developmental challenges with the purpose of augmenting development, or for preventive purposes for those parents that already have children with ASD. In comparison, a study of parents of children with fragile X (FX) found that 59% agreed that the FX-test influenced their decision to have another child [38]. It is surprising that only 24% agree that CGT is important for family planning (Table 2) when 40% would do a CGT if it enabled them to prevent having further children with ASD (Table 2). According to the World Health Organization (WHO), “family planning allows people to attain their desired number of children and determine the spacing of pregnancies. It is achieved through use of contraceptive methods and the treatment of infertility” [53]. This is also the official standard use of the term in Norway. However, the term may have been interpreted as very different from preventive measures, perhaps because this was addressed explicitly in another questionnaire statement. Family planning may have been interpreted as a question of organizing the family life and about interventions in the parents’ home as almost every child and teenager with ASD in Norway lives with their parents. Regarding genetic counselling and reproductive issues, this may warrant a focus on experienced and knowledgeable parents and collaboration between them and professionals, in addition to the current predominance of a “teaching model” that may not be optimal in supporting patients to make decisions relevant to their health care [54]. These results also seem to underscore the need for recurrence risk counselling.

Ethical concerns regarding genetic testing in a reproductive setting may vary with cultural, religious and societal value bases both in parents and professionals [55]. A collaborative climate that respects the autonomy of individuals and the values of the communities and groups to which they belong seems necessary [56]. It is also important to cover these ethical issues further than the scope of the present study, which may be limited by empirical foundations for such inquiries. Relevant issues for ethical inquiries include how society values people with ASD and their parents. Advocates for ASD fear the possible outcomes of prenatal genetic testing or treatment. They argue that aggressively seeking interventions devalues ASD traits and tendencies [42], and adults with ASD fear that people with these traits will eventually be eliminated through prenatal genetic testing and selective abortion [42].

Three quarters of our sample agreed that CGT is relevant for intervention planning. This seems in line with results from a study of the impact of CMA on clinical management [57]. Treatment plans targeting associated medical conditions and planned additional assessments were directly impacted for more than 40% of individuals with neurodevelopmental disorders, including ASD [58,59]. Furthermore, the current parents’ views seem in line with recent findings that genetic testing may shorten the ‘diagnostic odyssey’ and provide a causal explanation, indicate a recurrence risk and enable access to appropriate early interventions and tailoring the care of affected individuals [8,32,59,60]. Interestingly, a recent study summarizing the results of over four years of real-world clinical ultra-high resolution CMA testing optimized for neurodevelopmental disorders found direct correlation between a higher rate of detected abnormalities and age in the ASD cohort, which suggests that earlier use of CMA and perhaps other genetic testing methods may be important for early intervention [9].

Overall, characteristics of the parents or their children seem to have only small influences on the informants’ attitudes toward CGT of ASD. This underscores the general positive attitude toward the use of molecular genetic technology in this group of patients. Some differences were, however, present in parents of children with ASD. Whether the child had autism or Asperger syndrome influences the attitudes. Parents of children with autism are more optimistic about the clinical opportunities of genetic testing for ASD than parents of children with Asperger syndrome by rating the positive effects higher and the negative effects lower. The difference between Asperger syndrome and autism may be regarded as difference in disease severity. In contrast, no association between various FX severities and opinions about screening in caregivers have been found [61].

The stigma attached to mental illness is claimed to always lead to stress through discrimination in academic settings, in work, and in public, although often unintended [41]. For instance, in a future case of neonatal genetic screening for ASD, a child with a susceptibility genotype whose behavioral presentation is considered “odd” may be stigmatized as “autistic” despite not meeting the diagnostic criteria. Conversely, a child with a susceptibility genotype may be excused for “inappropriate” behavior [21]. In either case, less fortunate interventions or treatments may be initiated. Parents are also in danger of being blamed for their children’s condition when the condition is inherited. It is important to avoid terminology that may be misused and cause unjust stigmatizing and blaming, as happened with Kanner’s early characterization of parents [42]. If the ethical implications of genetic identities become uncertain as research gains more knowledge about how genes and environments interrelate [42], it is clear that empirical studies of attitudes, hopes and fears of all stakeholders, at pace with the technological advancements, are necessary.

### Limitations

There is limited knowledge about the attitudes toward CGT among parents of children with ASD. Although the current study provides important new information about the attitudes toward CGT for ASD and collected information from a large numbers of parents, it has limitations. One limitation is a lack of comprehensive demographic information of the participants, and little information about disease severity or genetic origin of the ASD in their children. Our findings are also limited to opinions about the specific items of positive and negative effects stated in our questionnaire. The lack of explicit questions about attitudes toward prenatal diagnosis and the related issues of stigma and offence of such practice is also a limitation. The low number of parents that think CGT should be offered during pregnancy may indicate that this is an issue that should have been addressed in more detail.

Another potential limitation is that the previously untested questionnaire was inspired from a questionnaire about malignant melanoma, which is a disease quite different from ASD. Malignant melanoma is a deadly skin cancer disease while ASD is a disability. There are important differences between what is perceived as disease and what is perceived as disability. Disabilities, and particularly those involving mental capacities, raise existential issues of identity in a sense that issues of diseases do not. To determine identities through genetic testing is more controversial than to determine diseases. This difference may have made the questions generated from malignant melanoma studies less suitable for capturing attitudes toward CGT for ASD. A further limitation is that the questionnaire did not specify whether the questions concerned testing parents or testing the child, where it could mean either, which makes the accuracy of some of our findings uncertain.

It is also possible that our sample is biased by being recruited from an interest organization. Our large sample may not be representative of attitudes of parents of children with ASD in general. Despite it being the only ASD organization in Norway, with a high number of members, a significant proportion of parents of children with ASD are not members of the interest organization.

## 4. Materials and Methods

### 4.1. Participants

In collaboration with the Norwegian patient organization for ASD “Autism Society Norway”, a questionnaire was sent by email or post to all of the parent members of the organization. The organization has no formal requirement for membership of parents, but 3539 members were registered as parents to one or more children with ASD. In order to reach as many as possible, two approaches were used. Members registered with email addresses were contacted by email, which contained a link to a web-based Response Form Questionnaire from the University of Oslo. Members without email addresses received paper mail with the questionnaire and a prepaid return envelope. Email reminders were sent to those not responding, while no paper mail reminders were sent. Answers were anonymous. A total of 3539 invitations were sent, *n* = 1990 by email and *n* = 1549 by paper letters. A total of *n* = 1455 responded (41%), *n* = 917 (46%) through email, and *n* = 538 (35%) by paper mail. The survey was closed after two reminders. No financial incentive or other rewards were provided.

### 4.2. Questionnaire

The questionnaire investigated attitudes toward clinical use of a potential genetic test for ASD. It was emphasized that, currently, a predictive genetic test for ASD is not available. The participants were asked to imagine the existence of a clinical test that could say “something” about the risk of ASD.

Some of the questions concerning attitudes were based on a survey of attitudes toward genetic research in families with a risk of malignant melanoma [62], others were composed and tested by our multi-professional team and selected representatives of the target group. One of the questions was also in line with a qualitative study of awareness and attitudes among parents of children with ASD [25]. The questionnaire as a whole has not been previously tested and the overall psychometric properties are unknown. Although difficult to establish, the validity seems high at face value. Our explorations of how responder characteristics influence the ratings provide some indications of the validity of the questionnaire. The answers were given on a 5-point Likert scale with alternatives ”strongly disagree”, “disagree”, “neither disagree or agree”, “agree” and “strongly agree”, in addition to “Don´t know” or “Have no meaning” options [63].

The questions concerning attitudes were part of a questionnaire with 45 items in four sections. It took 10–15 min to complete the questionnaire. The first section of the questionnaire asked about age and gender of the parents. The second section asked about the parents’ firstborn child with ASD diagnosis, the child´s ASD diagnosis (infantile autism or Asperger syndrome), the child´s age when first diagnosed with ASD, and the child´s comorbid somatic and psychiatric diagnoses. It was not asked how many children with ASD the participants have. International Statistical Classification of Diseases and Related Health Problems (ICD) criteria are used in Norway. The third section of the questionnaire asked about experiences with the specialist health services. The fourth section asked about attitudes toward genetic research and CGT. The questions about attitudes toward genetic research have been previously reported [36].

With a total of *n* = 1455 members responding, we estimate the response rate to be approximately 50%. Because the information to be collected was anonymized, the Regional Committee for Medical and Health Research Ethics (REC) concluded that the study did not require REC approval.

### 4.3. Data Handling and Analyses

SPSS version 23 (IBM, Armonk, NY, USA) was used for statistical analyses. The paper responses were scanned and read using ABBYY FlexiCapture 10 (ABBYY Europe, Munich, Germany) and subsequently imported into a SPSS file, while the online responses were imported directly.

First, descriptive statistics were performed. Percentages of agreement and disagreement to the attitude statements were obtained by dichotomizing the Likert-scale variables in the following way: Scores 1 (“Totally disagree”) and 2 (“Somewhat disagree”) were combined to a “disagree”—score, scores 4 (“Somewhat agree”) and 5 (“Totally agree”) were combined to an “agree”—score. Score 3 (“neither/nor”) was merged with the “have no meaning”—and the “don’t know”—scores in order to form an “NA”—score labelling the “no distinct negative or positive attitude available”.

Forced entry linear regression analyses were conducted both on the original 5-point attitude variables and on the binary attitude variables [64] concerning management of CGT to analyze the effect of respondent age and gender, child age, child gender, and child ASD diagnose on the attitude items. Due to multiple comparisons, we used Bonferroni corrected significance thresholds, setting the significance limits to *p* = 0.01 per test (0.05/5 tests). Cases with missing values were excluded listwise in the regression analyses and deleted on an analysis-by-analysis basis in the independent sample *t*-tests.

## 5. Conclusions

Parents of children with ASD seem to be clearly positive toward CGT. Only one of ten is opposed to CGT and the reason is mainly skepticism about the possible benefits, not because they fear possible harm. This suggests that parents of children with ASD seem well informed, have a practical attitude to CGT, and have similar attitudes as health care personnel and ASD experts.

Few parents seem interested in prenatal diagnosis and family planning. They seem rather to prefer CGT when a child shows behavioral or developmental challenges with the purpose of augmenting development, or for preventive purposes for those that already have children with ASD. Further research is necessary in order to draw direct clinical implications of these results. However, the current results may form the basis for such testing procedures in the future when they will be more informative. It is important to avoid initiating premature CGT procedures.

## Figures and Tables

**Table 1:** Demographics: Responding parents and their children with ASD.

Responding Parents *n* = 1444 ^a^	Mean Age, (SD), Age Range, [count], %
Age: mean (SD) {range}	46.7 (8.6) {22–87}
Children with ASD of the responding parents: [count]	[1432] ^b^
Age of the children with ASD: mean (SD) {range}	16.5 (7.6) {3–58}
Male gender of the children with ASD ^c^	80.5%
ASD sub-diagnoses of the children with ASD of the responding parents ^d^	
Asperger syndrome	49.3%
Infantile autism	37.6%
Other ASD	13.1%

Missing information: ^a^ *n* = 11; ^b^ *n* = 23; ^c^ *n* = 29; ^d^ *n* = 39; ASD: Autism spectrum disorder, SD: Standard deviation.

**Table 2:** Parents’ opinions on possible effects of CGT for ASD (*n* = 1455).

**Positive Effects**	**Agree**
Causal Explanation ^a^	76.4%
I would only take a genetic test if it could help explain the cause of ASD in me or in my child.	
Intervention Planning ^b^	74.8%
Early diagnosis based on a genetic test will improve the possibilities of planning for good interventions and facilitations.	
Treatment Relevance ^c^	53.1%
I would only take a genetic test if it has a direct relevance for treatment or intervention.	
Recurrence Prevention ^d^	40.0%
I would take a genetic test if it gave me the possibility to prevent having more children with ASD.	
Family Planning ^e^	24.1%
Genetic testing is important for family planning.	
**Negative Effects**	**Agree**
Insurance Discrimination ^f^	66.8%
A genetic test showing ASD risk will cause discrimination from insurance companies.	
Parental Concern ^g^	56.1%
Early diagnosis based on a genetic test will increase the parents’ concern for the future health and development of the child.	
Family Conflicts ^h^	48.9%
Genetic testing may cause family conflicts.	

Missing information: ^a^ *n* = 33; ^b^ *n* = 74; ^c^ *n* = 42; ^d^ *n* = 36; ^e^ *n* = 38; ^f^ *n* = 68; ^g^ *n* = 71; ^h^ *n* = 76; CGT: Clinical genetic testing; ASD: Autism spectrum disorder.

**Table 3:** Parents’ opinions on management of CGT (*n* = 1330).

**Who Should Be Offered Testing**	**Agree**
Parents of children with ASD	64.9%
Those already having a child with ASD	
Doubting able to care	15.6%
Those doubting ability to care for a child with ASD	
Worried about the fetus	14.7%
Those anxious/worried that there is something wrong with the fetus	
No pregnant women	11.3%
All pregnant women	9.5%
**When Should Testing Be Offered**	**Agree**
Development deviates	47.7%
When a child shows behavioral or developmental difficulties	
Before pregnancy	35.6%
During pregnancy	8.6%
Immediately after birth	8.1%

CGT: Clinical genetic testing; ASD: Autism spectrum disorder.

**Table 4:** Parents in favor versus parents opposed CGT.

Possible Positive Effects	Agree N	In Favor of CGT	Opposed CGT	MD	Sig.
Causal Explanation	1422	80.7%	30.9%	1.9	**
I would take a genetic test only if it could help explain the cause of ASD in me or in my child.					
Recurrence Prevention	1419	43.3%	4.9%	1.8	**
I would take a genetic test if it gave me the possibility to prevent getting more children with ASD.					
Intervention Planning	1381	78.6%	35.0%	1.4	**
Early diagnosis based on a genetic test will improve the possibilities of planning for good interventions and facilitations.					
Family Planning	1417	26.3%	0.8%	1.4	**
Genetic testing is important for family planning.					
Treatment Relevance	1413	54.1%	41.8%	0.7	**
I would take a genetic test only if it has directly treatment-relevant consequences for me or my child.					
Possible negative effects					
Family Conflicts	1379	47.3%	66.7%	-0.9	**
Genetic testing may cause family conflicts.					
Parental Concern	1384	54.9%	68.9%	-0.7	**
Early diagnosis based on a genetic test will increase the parents’ concern for the future health and development of the child.					
Insurance Discrimination	1387	66.0%	74.4%	-0.4	*
A genetic test showing ASD risk will cause discrimination from insurance companies.					

CGT: Clinical genetic testing; ASD: Autism spectrum disorder; MD: Difference in means; Sig.: Significance probability; ** *p* < 0.001, * *p* < 0.01.

## Supplementary Materials

Supplementary materials can be found at www.mdpi.com/1422-0067/18/5/1078/s1.

## Abbreviations

ASD	Autism spectrum disorder
CGT	Clinical genetic testing
CMA	Chromosomal microarray
CNV	Copy number variant
DS	Down syndrome
ESHG	European Society of Human Genetics
FX	Fragile X
FXS	Fragile X syndrome
ICD	International Statistical Classification of Diseases and Related Health Problems
REC	Regional Committee for Medical and Health Research Ethics
VPHI	Voluntary private health insurance
WHO	World Health Organization
