# Supplementary material for: Parents’ Attitudes toward Clinical Genetic Testing for Autism Spectrum Disorder—Data from a Norwegian Sample
Source: Int J Mol Sci. 2017 May 18;18(5):1078. doi: 10.3390/ijms18051078 (PMC5454987; doi:10.3390/ijms18051078)
Supplement: Supplementary file 1 [file ijms-18-01078-s001.pdf]

Supplementary Materials: Parents' Attitudes toward Clinical Genetic Testing for Autism Spectrum Disorder—Data from a Norwegian Sample

Jarle Johannessen, Terje Nærland, Sigrun Hope, Tonje Torske, Anne Lise Høyland, Jana Strohmaier, Arvid Heiberg, Marcella Rietschel, Srdjan Djurovic and Ole A. Andreassen

Table S1a. Regression models summary of possible positive effects of clinical genetic testing.

	**Causal Explanation**				**Intervention Planning**				**Treatment Relevance**			
Factors	B	Sig.	99% CI		B	Sig.	99% CI		B	Sig.	99% CI	
Constant	4.755	0.000	3.790	5.721	4.286	0.000	3.340	5.231	3.061	0.000	1.841	4.281
Parents' gender parent	−0.183	0.062	−0.435	0.069	−0.144	0.133	−0.390	0.103	−0.025	0.841	−0.341	0.292
Parent's age	0.002	0.771	−0.017	0.021	0.004	0.526	−0.014	0.023	−0.002	0.865	−0.025	0.022
Gender of children with ASD	−0.022	0.820	−0.267	0.223	0.003	0.970	−0.233	0.239	0.068	0.568	−0.239	0.375
Age of children with	0.007	0.372	−0.014	0.028	−0.005	0.536	−0.025	0.016	0.008	0.462	−0.019	0.034
ASD diagnose of children	−0.272	0.000	−0.470	−0.075	−0.002	0.975	−0.193	0.189	0.188	0.051	−0.061	0.437
Model´s R^2	0.018				0.003				0.005			
Model´s p	0.001				0.638				0.329			
Model´s N	1109				1050				1055			

B = Unstandardized Beta coefficients, Sig. = significant level and 99% CI = 99% confidence interval for B for each factor entered in the regression models with statements of possible positive effects as outcome. ASD: Autism Spectrum Disorder.

	**Recurrence Prevention**				**Family Planning**			
Factors	B	Sig.	99% CI		B	Sig.	99% CI	
Constant	4.529	0.000	3.226	5.831	2.636	0.000	1.510	3.763
Parents' gender	−0.522	0.000	−0.861	−0.182	−0.108	0.345	−0.402	0.186
Parents' age	0.009	0.360	−0.016	0.034	0.023	0.007	0.001	0.045
Gender of children with ASD	−0.027	0.833	−0.358	0.304	−0.189	0.082	−0.468	0.091
Age of children with ASD	0.012	0.288	−0.017	0.041	−0.008	0.368	−0.033	0.016
ASD diagnose of children	−0.801	0.000	−1.068	−0.534	−0.494	0.000	−0.720	−0.267
Model´s R^2	0.081				0.044			
Model´s p	0.000				0.000			
Model´s N	1047				1090			

B = Unstandardized Beta coefficients, Sig. = significant level and 99% CI = 99% confidence interval for B for each factor entered in the regression models with statements of possible positive effects as outcome. ASD: Autism Spectrum Disorder.

Table S1b. Regression models summary of possible negative effects of clinical genetic testing.

	**Insurance Discrimination**				**Parental Concern**				**Family Conflicts**			
Factors	B	Sig.	99% CI		B	Sig.	99% CI		B	Sig.	99% CI	
Constant	3.814.	0,000	2.840	4,789	2.550	0.000	1.444	3.657	2.990	0.000	1.922	4.057
Parents' gender	0.064	0.515	−0.189	0.316	0.061	0.584	−0.228	0.350	0.062	0.562	−0.214	0.339
Parents' age	0.006	0.429	−0.013	0.025	0.021	0.011	0.000	0.043	0.010	0.190	−0.010	0.031
Gender of children with ASD	−0.011	0.910	−0.250	0.229	0.026	0.811	−0.251	0.302	0.023	0.823	−0.239	0.284
Age of children with ASD	−0.016	0.053	−0.037	0.005	−0.020	0.028	−0.044	0.004	−0.018	0.048	−0.040	0.005
ASD diagnose of children	0.199	0.009	0.003	0.395	0.080	0.359	−0.144	0.304	0.107	0.202	−0.109	0.322
Model´s R^2	0.011				0.007				0.005			
Model´s p	0.055				0.219				0.404			
Model´s N	942				1058				977			

B = Unstandardized Beta coefficients, Sig. = significant level and 99% CI = 99% confidence interval for B for each factor entered in the regression models with statements of possible negative effects as outcome. ASD: Autism Spectrum Disorder.

Table S2a. Regression models summary of who to offer genetic testing (N = 1096).

	Parents of children with ASD				Doubting able to care				Worried about the fetus			
Factors	B	Sig.	99% CI		B	Sig.	99% CI		B	Sig.	99% CI	
Constant	0.786	0.000	0.414	1.158	0.224	0.044	−0.063	0.512	0.352	0.001	0.079	0.626
Parents' gender	−0.037	0.320	−0.134	0.060	−0.054	0.062	−0.129	0.021	−0.129	0.000	−0.200	−0.058
Parents' age	0.003	0.303	−0.004	0.010	0.002	0.409	−0.004	0.007	0.003	0.143	−0.002	0.008
Gender of children with ASD	−0.034	0.352	−0.127	0.060	−0.003	0.911	−0.075	0.069	0.013	0.628	−0.056	0.082
Age of children with ASD	0.001	0.687	−0.007	0.009	0.000	0.951	−0.006	0.006	0.000	0.927	−0.006	0.006
ASD diagnose of children	−0.122	0.000	−0.198	-0.046	−0.031	0.173	−0.089	0.028	−0.080	0.000	−0.135	-0.024
Model´s R^2	0.022				0.008				0.044			
Model´s p	0.000				0.109				0.000			

B = Unstandardized Beta coefficients, Sig. = significant level and 99% CI = 99% confidence interval for B for each factor entered in the regression models with who to offer genetic testing statements as outcome. ASD: Autism Spectrum Disorder.

	No Pregnant				All pregnant			
Factors	B	Sig.	99% CI		B	Sig.	99% CI	
Constant	0.030	0.753	−0.219	0.280	0.516	0.000	0.299	0.733
Parents' gender	0.063	0.013	−0.003	0.128	−0.146	0.000	−0.202	−0.089
Parents' age	−0.002	0.320	−0.007	0.003	−0.004	0.021	−0.008	0.000
Gender of children with ASD	0.006	0.813	−0.057	0.068	0.014	0.522	−0.041	0.068
Age of children with ASD	0.000	0.838	−0.006	0.005	0.003	0.155	−0.002	0.007
ASD diagnose of children	0.039	0.050	−0.012	0.089	−0.031	0.074	−0.075	0.014
Model´s R^2	0.015				0.045			
Model´s p	0.006				0.000			

B = Unstandardized Beta coefficients, Sig. = significant level and 99% CI = 99% confidence interval for B for each factor entered in the regression models with who to offer genetic testing statements as outcome. ASD: Autism Spectrum Disorder.

Table S2b. Regression models summary of when to offer genetic testing (N = 1096).

	Development deviates				Before pregnancy				During pregnancy			
Factors	B	Sig.	99% CI		B	Sig.	99% CI		B	Sig.	99% CI	
Constant	0.596	0.000	0.204	0.987	0.577	0.000	0.206	0.947	0.306	0.000	0.082	0.530
Parents' gender	−0.035	0.376	−0.137	0.067	−0.068	0.068	−0.165	0.028	−0.077	0.001	−0.135	−0.018
Parents' age	−0.004	0.215	−0.011	0.004	0.001	0.606	−0.006	0.009	−0.001	0.686	−0.005	0.004
Gender of children with ASD	0.033	0.382	−0.065	0.132	0.002	0.957	−0.091	0.095	0.003	0.885	−0.053	0.059
Age of children with ASD	0.001	0.812	−0.008	0.009	−0.001	0.826	−0.009	0.007	0.001	0.539	−0.004	0.006
ASD diagnose of children	0.046	0.138	−0.034	0.126	−0.106	0.000	−0.182	−0.031	−0.043	0.015	−0.089	0.002
Model´s R^2	0.006				0.018				0.017			
Model´s p	0.306				0.002				0.002			

B = Unstandardized Beta coefficients, Sig. = significant level and 99% CI = 99% confidence interval for B for each factor entered in the regression models with when to offer genetic testing statements as outcome. ASD: Autism Spectrum Disorder.

	Immediately after birth			
Factors	B	Sig.	99% CI	
Constant	0.158	0.053	−0.053	0.368
Parents' gender	−0.029	0.180	−0.083	0.026
Parents' age	0.000	0.857	−0.004	0.004
Gender of children with ASD	0.020	0.335	−0.033	0.073
Age of children with ASD	−0.002	0.160	−0.007	0.002
ASD diagnose of children	−0.015	0.362	−0.058	0.028
Model´s R^2	0.008			
Model´s p	0.116			

B = Unstandardized Beta coefficients, Sig. = significant level and 99% CI = 99% confidence interval for B for each factor entered in the regression models with when to offer genetic testing statements as outcome. ASD: Autism Spectrum Disorder.
